# Nitrogen rate impacts on tropical maize nitrogen use efficiency and soil nitrogen depletion in eastern and southern Africa

**DOI:** 10.1007/s10705-020-10049-x

**Published:** 2020-02-13

**Authors:** Heather R. Pasley, James J. Camberato, Jill E. Cairns, Mainassara Zaman-Allah, Biswanath Das, Tony J. Vyn

**Affiliations:** 1 Agronomy Department, Purdue University, 915 W. State Street, West Lafayette, IN 47907 USA; 2 International Maize and Wheat Improvement Centre (CIMMYT), Harare, Zimbabwe; 3 International Maize and Wheat Improvement Centre (CIMMYT), Nairobi, Kenya

**Keywords:** Maize hybrids, Nitrogen fertilizer, Soil nitrogen depletion, Nitrogen use efficiency, Africa

## Abstract

Sub-Saharan Africa is facing food security challenges due, in part, to decades of soil nitrogen (N) depletion. Applying N fertilizer could increase crop yields and replenish soil N pools. From 2010 to 2015, field experiments conducted in Embu and Kiboko, Kenya and Harare, Zimbabwe investigated yield and N uptake response of six maize (*Zea mays* L.) hybrids to four N fertilizer rates (0 to 160 kg N ha^−1^) in continuous maize production systems. The N recovery efficiency (NRE), cumulative N balance, and soil N content in the upper 0.9 m of soil following the final harvest were determined at each N rate. Plant and soil responses to N fertilizer applications did not differ amongst hybrids. Across locations and N rates, NRE ranged from 0.4 to 1.8 kg kg^−1^. Higher NRE values in Kiboko and Harare occurred at lower post-harvest soil inorganic N levels. The excessively high NRE value of 1.8 kg kg^−1^ at 40 kg N ha^−1^ in Harare suggested that maize hybrids deplete soil inorganic N most at low N rates. Still, negative cumulative N balances indicated that inorganic soil N depletion occurred at all N rates in Embu and Harare (up to − 193 and − 167 kg N ha^−1^, respectively) and at the 40 kg N ha^−1^ rate in Kiboko (− 72 kg N ha^−1^). Overall, maize N uptake exceeded fertilizer N applied and so, while yields increased, soil N pools were not replenished, especially at low total soil N levels (< 10,000 kg N ha^−1^ in top 0.9 m).

**Electronic supplementary material:**

The online version of this article (10.1007/s10705-020-10049-x) contains supplementary material, which is available to authorized users.

## Introduction

Maize cropping systems in Kenya have been reported to deplete the soil an average of 42 kg N ha^−1^ annually (Smaling et al. 1993). In Zimbabwe, the annual depletion rates are purportedly lower than those of Kenya at around 30 kg N ha^−1^ (Henao and Baanante 2006). This difference in depletion is in part due to the one growing season per year in Zimbabwe yielding an average 3 t ha^−1^ year^−1^ less grain than the combined yields of 2 growing seasons per year in Kenya between 1961 and 2016 (FAOSTAT 2019). Although soil erosion plays a large role in soil degradation, Drechsel et al. (2001) found that the average N and K balances in sub-Saharan Africa (SSA) would be negative even in scenarios where no erosion was occurring. Since the majority of soils in SSA have large soil K reservoirs, the short-term agronomic impact of K depletion is most likely negligible (Vanlauwe and Giller 2006). On the other hand, a negative N balance in SSA’s low N environments is likely to have negative effects on crop production. Moreover, the risk of potential negative effects has increased in conjunction with crop intensification aimed at meeting the food needs of SSA’s growing population (Drechsel et al. 2001).

One potential solution for mitigating the depletion of soil N is the application of inorganic N fertilizer. The average rate of inorganic N fertilizer applied in SSA, however, is only 5–7 kg N ha^−1^ in 2010/11 and is expected to only reach ~ 12 kg N ha^−1^ in 2020 (Sheahan and Barrett 2017; AfricaFertilizer.org 2019). This greatly contrasts with the 2013 global average of 74 kg N ha^−1^ (Lu and Tian 2017). Few field experiments in SSA to date, however, have quantified soil N depletion at inorganic fertilizer N rates less than 100 kg N ha^−1^ (Akintoye et al. 1999; Nyamangara et al. 2003; Oikeh et al. 2003) and no previous study, to our knowledge, has examined N depletion at inorganic fertilizer N rates less than 60 kg N ha^−1^. While older studies often referenced the high cost of fertilizer as a major reason for the low application rates, more recent work has focused on challenges associated with a lack of access to fertilizers and on low potential profits from any fertilizer input due to the variability in yield response to N (Stocking 1988; Larson and Frisvold 1996; Crawford et al. 2003; Sileshi et al. 2010; Vanlauwe et al. 2014; Kihara et al. 2016; Njoroge et al. 2017; Maman et al. 2018). Both nutrient and water limitations in maize have been cited in many studies as primary causes of SSA’s low yields (Zingore et al. 2015; Kihara et al. 2016; Johansson et al. 2016).

While organic fertilizer and crop residue have been proposed as being more affordable sources of nutrients, they do not currently provide enough nutrients to meet the needs of maize grown on small-holder subsistence farms, much less the needs of maize in an intensified production scenario (Bationo et al. 1998; Franzluebbers 2002; Mtangadura et al. 2017). In addition, crop residues are also used as feed and, therefore, are often not available for soil fertility improvement. It is crucial, therefore, to determine how applying moderate (< 100 kg N ha^−1^) and low (< 60 kg N ha^−1^) amounts of N impacts grain yield, especially in a continuous maize cropping system, which is more dependent on higher N input levels than maize in rotation and is commonly utilized in eastern and southern Africa (Tully et al. 2016).

Maize response to N management in any environment must always be understood in the context of the actual genotypes employed. Hybrids account for around 80% of the maize cultivars planted in Kenya and around 95% in Zimbabwe. Many farmers plant more than one maize cultivar on their land (up to 5 in Kenya and up to 4 in Zimbabwe) (Fisher et al. 2015). In order to test the viability of N fertilizer as a potential solution for the yield gap and soil N depletion in SSA, it is essential to investigate the yield response and N recovery efficiency (additional plant N content per unit N applied) of a variety of hybrids: both hybrids that are widely popular in the targeted regions as well as those marketed as superior to currently adopted hybrids (Jama et al. 2017).

This study, therefore, quantified apparent soil N depletion across the rooting zone after 5 to 9 seasons in continuous maize cultivation in three low N sites varying in weather conditions and soil properties. We evaluated the capacity of inorganic N fertilizer applied consistently at multiple rates to mitigate this depletion.

## Materials and methods

### Site description

Three field experiments [Embu, Kenya (00°31′S 37°29′E); Kiboko, Kenya (02°13′S 27°42′E); and Harare, Zimbabwe (17°43′S 31°5′E)] were established in 2010. These sites were under continuous maize cultivation for a total of 9 seasons in Embu, 7 seasons in Kiboko, and 5 in Harare with 2 seasons a year in the Kenya sites and 1 season a year in Zimbabwe.

Embu’s soils were Humic Nitisols, Kiboko’s soils were Acri-Rhodic Ferrassols, and Harare’s soils were Ferric Luvisols. These sites were selected to capture some of the variability in soils and agroecosystems in SSA. In the 5 years preceding the initiation of this experiment, continuous maize (in Embu and Harare) and sorghum (*Sorghum bicolor* L.) (in Kiboko) were cultivated continuously without any inorganic or organic inputs to simulate the N depleted conditions of the average small-holder farm in SSA. More details about the site conditions can be found in Supplementary Table 1 and in Pasley et al. (2019).

### Management and research design

The experiment was a split-plot design with 4 replications in which N rate was the main plot and hybrid the sub-plot. The locations of the main and sub-plots were randomized the first season, but were fixed thereafter.

Four N fertilizer rates (0, 30, 60, 90 kg N ha^−1^ in Embu and 0, 40, 80, 160 kg N ha^−1^ in Kiboko and Harare) were applied in two applications, the first 30% at planting mixed into the seed hill and the remaining 70% broadcast 5–6 weeks after planting. The fertilizer source was calcium ammonium nitrate in Embu and Kiboko and ammonium nitrate in Harare. At the time of the main N application, 20 kg P ha^−1^ was broadcast applied as triple superphosphate. All above-ground biomass was routinely removed at harvest in Embu and Harare, while 1/3 of the post-harvest stover biomass was returned in Kiboko. More plot management details can be found in Supplementary Table 2 and in Pasley et al. (2019).

Six maize hybrids, consisting of commercially available and drought-tolerant CIMMYT hybrids, were selected for each site (for more details, see Supplementary Table 3 and Pasley 2018). Commercial hybrids were selected based on their prevalence among farmers in the targeted countries. For instance, in 2013/14, one hybrid we used (Duma43) was planted on 20% of the land area, more than any other individual hybrid in Kenya, while Duma43 and two other hybrids we used (SC513 and PAN413) were the most widely grown hybrids in Zimbabwe with SC513 alone planted on ~ 40% of the maize area in the country (Fisher et al. 2015). CIMMYT hybrids were selected for their water-use efficiency as evaluated during controlled drought studies as part of the Water Efficient Maize for Africa project (Beyene et al. 2015). Final plant population densities were uniform across hybrids and years within a site, and averaged 53,300, 44,400, and 66,700 plants ha^−1^ in Embu, Kiboko, and Harare, respectively (Supplementary Table 2).

In this paper, grain yield is reported at 15.5% moisture whereas grain and plant biomass are given at 0% moisture. The following calculations were used to quantify N efficiency:

1 $${\text{N}}\;{\text{Recovery}}\;{\text{Efficiency }}\left( {\text{NRE}} \right) = \frac{{{\text{Total}}\;{\text{Plant}}\;{\text{N}}\;{\text{at}}\;{\text{R}}6_{{{\text{N}}\;{\text{Rate}}}} - {\text{Total}}\;{\text{Plant}}\;{\text{N}}\;{\text{at}}\;{\text{R}}6_{{{\text{0}}\;{\text{kg}}\;{\text{N}}\;{\text{ha}}^{ - 1} }} }}{{{\text{N}}\;{\text{Rate}}\;{\text{Applied}}}}$$

2 $${\text{Fertilizer}}\;{\text{N}}\;{\text{Balance}} = {\text{N}}_{\text{Applied}} \left( {{\text{kg ha}}^{ - 1} } \right) - {\text{Plant}}\;{\text{N}}_{\text{removed}} \left( {{\text{kg ha}}^{ - 1} } \right)$$

Both equations were used to quantify the crop’s demand for N and how well the application of N fertilizer mitigated soil N depletion. In Kiboko, where 1/3 of the stover biomass was returned to the soil post-harvest, only 2/3 of the total plant N was used to calculate plant N removal in the fertilizer N balance Eq. (2). In addition to the fertilizer N balance, the soil–plant N balance approach can be used to quantify possible legacy soil N from fertilizer applications in earlier seasons and allows for a hybrid’s recovery of N to be related to the hybrid’s response to the naturally available soil N reservoirs that were not applied as fertilizer.

### Field sampling and laboratory analyses

The plant sample collection schedule is outlined in detail in Supplementary Table 2, but is described here in brief. Every season, the center 13.5 m^2^ area in each plot in Embu and Harare and the center 7.5 m^2^ area in each plot in Kiboko were harvested for grain yield estimation (differences in the harvested area reflected differences in the land availability at each site and, thus, the plot sizes). At harvest of the ninth season in Embu (2015SR), the fifth and sixth seasons in Kiboko (2013LR and 2014SR), and the fifth season in Harare (2014/15), 6 to 10 plants were sampled from the center of each plot at harvest and partitioned into grain and stover biomass. A Retsch SS MM200 Ball Mill Plant was used to grind these partitioned plant tissue samples to < 100 µm. A flash 2000 CHN Analyzer (ThermoFisher Scientific Inc.) was used to analyze for total C and N concentrations in the plant samples via the combustion method (Etheridge et al. 1998).

Following the 2015 harvest in all sites, composite soil samples comprised of 5 or 15 soil cores were taken near the center of each plot (both in row and between row) to a depth of 0.9 m in 5 depth increments (0–0.15, 0.15–0.3, 0.3–0.45, 0.45–0.6, and 0.6–0.9 m). Fewer cores (5) were taken in Embu due to the excessively compacted nature of the soil. Soil samples were ground, sieved through a 2 mm screen, and sub-sampled before shipping to Purdue University for analyses. Bulk density for each depth increment was measured using intact cores (Blake 1965).

Chemical and physical analyses of soil were performed to measure non-N essential plant nutrients and other factors (texture, CEC, pH etc.) that have been found to enhance or limit nutrient/water availability in maize systems (see Supplementary Table 1; Pasley et al. 2019 for more details). Soil samples were extracted with a 1 M KCl solution (10:1) and analyzed for NO_3_-N and NH_4_-N using an AQ2 Discrete Analyzer (SEAL Analytical) (Keeney and Nelson 1982). A sub-sample was ground to 100 µm diameter using a Retsch SS MM200 Ball Mill and analyzed for total C and N using the combustion method (Bremner and Mulvaney 1982; Nelson and Sommers 1982). Carbonates in soils were negligible, thus soil organic matter (OM) was estimated using the total C concentration and a single factor estimation of OM to C conversion factor of 2:1 (Pribyl 2010).

### Meteorological data

Rainfall/temperature data (NASA 2020) were totaled/averaged for the season, the critical period (the time interval starting 2 weeks before anthesis and ending 2 weeks after silking), and for the grain filling period (estimated to begin 2 weeks after the end of the critical period and ending at harvest) (Supplementary Table 4; Pasley et al. 2019). An indeterminate amount of irrigation was applied in Harare and Kiboko to supplement rainfall throughout the season. In Embu, the plots were not irrigated and low rainfall levels resulted in dry growing conditions (Supplementary Table 4; Pasley et al. 2019).

### Statistical analysis

Data was analyzed using SAS 9.4 PROC Mixed ANOVA and differences in Least Squares Means were considered significant at *P *≤ 0.05. Seasons could not be pooled for plant data due to significant variance in residuals. The resulting least squares means were compared to a constant with a 2-tailed LSD ($\propto = 0.05$). The factors investigated were N rate and depth (for soil). Stover N was only measured in 1 or 2 seasons at each site. To calculate the cumulative plant N content and, thus cumulative N balance, total plant N content was estimated using the N harvest index which was calculated as grain N content per total plant N content, using measured grain N concentration data from each hybrid and N rate plot (N harvest index averaged 0.66 in Embu, 0.52 in Kiboko, and 0.59 in Harare).

Pearson Correlations were conducted to analyze the linear relationship between total soil N in the upper 0.9 m of soil and cumulative grain yield for all seasons (9 seasons in Embu, 7 seasons in Kiboko, 5 seasons in Harare) in each plot. The linear regression fit was selected, as the quadratic component was not significant (*P *> 0.05). The slopes of cumulative grain yield response to change in total soil N as affected by N rate were tested using linear regression.

## Results

### Soil characteristics

Soil at Embu and Harare was a clay/clay loam (Supplementary Table 1a and c, respectively) while at Kiboko soil was a sandy loam/sandy clay loam (Supplementary Table 1b). In the upper 0.15 m, OM, P and K levels and bulk density values were: Embu—5.8%, 24 mg P kg^−1^, 373 mg K kg^−1^, and 0.93 Mg m^−3^; Kiboko—2.6%, 107 mg P kg^−1^, 240 mg K kg^−1^, and 1.53 Mg m^−3^; Harare—2.6%, 21 mg P kg^−1^, 135 mg K kg^−1^, and 1.37 Mg m^−3^ (Pasley et al. 2019). These values suggest that Embu and Harare had an insufficient amount of soil P while Kiboko had an optimal P level (Heckman et al. 2006). All sites had optimal levels of K (Heckman et al. 2006).

### Grain yield and plant nitrogen dynamics

The data reported in this paper are averaged over all hybrids, but detailed hybrid data is reported in Pasley (2018). Unless otherwise noted, there were no significant interactions between hybrid, N rate, and/or depth effects for any of the plant–soil N dynamics reported in this paper.

Grain yield response to N fertilizer application rates was analyzed and used to calculate the agronomic optimal N rate for each site in Pasley et al. (2019). The following text is a summary of those findings to provide context for the results reported in this paper. Grain yield increased at the lowest rate of applied N each season at all sites, but yield plateaued at the low or medium N rate (30 or 60 kg N ha^−1^) in Embu and at the medium N rate (80 kg N ha^−1^) in Harare. Grain yield did not plateau in Kiboko within the range of N rates applied in any season. There was no hybrid by N rate interaction for grain yield at any site. Averaged over all hybrids, in the final season of the experiment, grain yield ranged from 3.6 to 5.2 t ha^−1^ in Embu, 4.0 to 7.6 t ha^−1^ in Kiboko, and 5.4 to 7.9 t ha^−1^ in Harare.

**Fig. 1:**
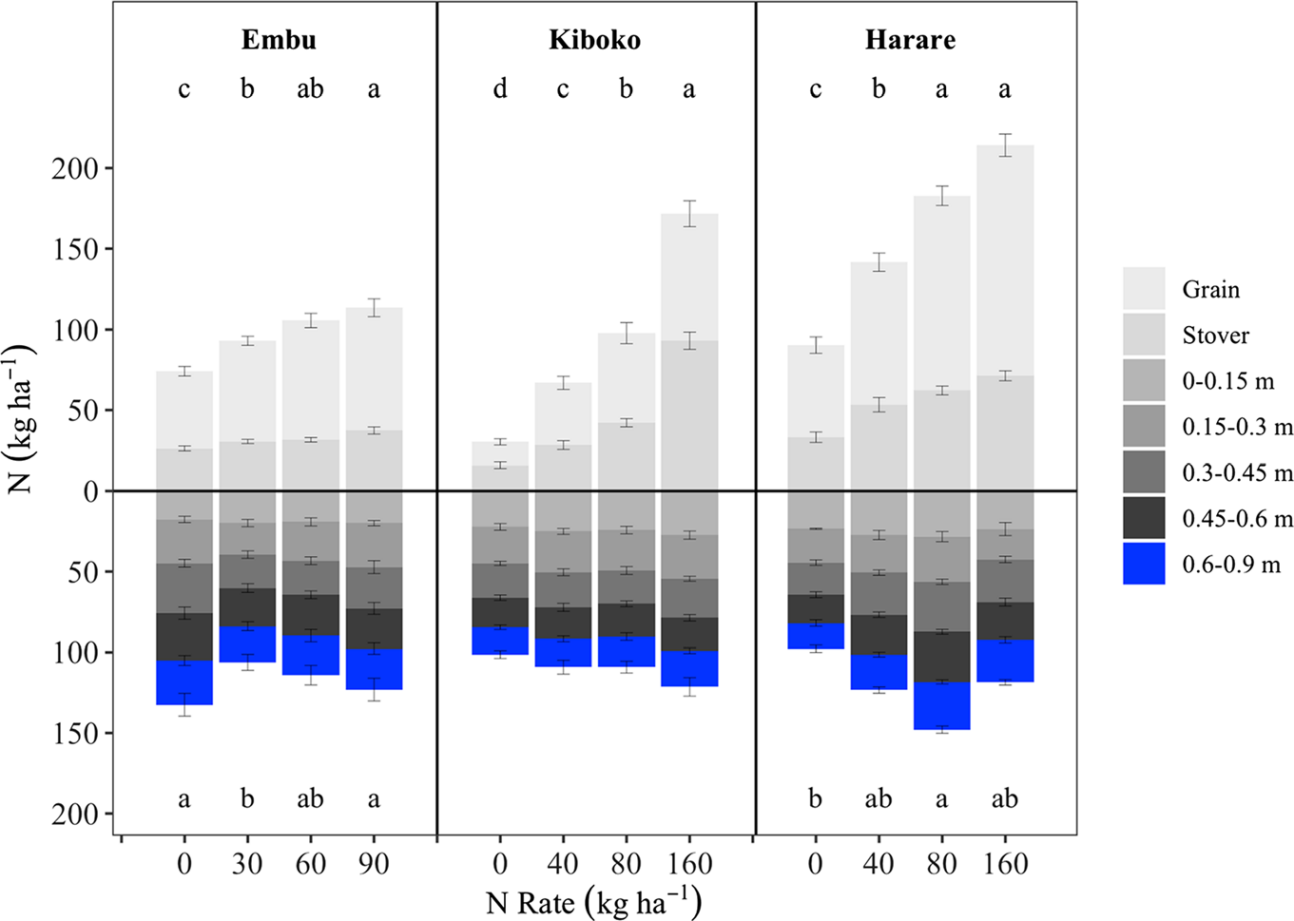
Aboveground plant N and soil inorganic N to a depth of 0.9 m in Embu, Kenya (post-season 2015SR), Kiboko, Kenya (2013LR for plant*; post-season 2015SR for soil), and Harare, Zimbabwe (post-season 2014/15). Letters denote differences among the total plant or total soil contents at different N rates averaged across 6 hybrids as assessed by LSD ($\propto = 0.05$). Where there are no letters, the difference was not significant. Error bars show the standard error of the mean. *The N rate effects on total plant N content in Kiboko in the 2014SR season were the same as in 2013LR (plant N content increased as N rate increased) and so the grain and stover data presented is the average of the two seasons

Total plant N content increased as N rate increased in all sites, but plateaued at the medium N rates in Embu and Harare (60 and 80 kg N ha^−1^, respectively) (Fig. 1). As was the case with grain yield, plant N content did not plateau within the applied N rate range in Kiboko. Plant N content did not differ consistently among hybrids from season to season at any site (Pasley 2018).

The NRE values were high, averaging 0.9 kg kg^−1^ across all applied N rates in Kiboko and at 1.5 kg kg^−1^ at the lowest two non-zero N rates in Harare (Table 1). There was no N rate effect on NRE in Embu or Kiboko. In Harare, NRE decreased from 1.8 to 0.69 kg kg^−1^ as N rate increased from 40 to 160 kg N ha^−1^.

**Table 1:** Effects of N rate on N recovery efficiency (NRE) and cumulative N balance (calculated using the average NHI value of 0.66 from Embu 2015SR season, 0.52 from Kiboko 2013LR and 2014SR seasons, and 0.57 from Harare 2014/15 season and grain N contents from each season) averaged across all hybrids

Site	Variable	Zero N	Low N	Medium N	High N
Embu	NRE (kg kg^−1^)		0.65	0.49	0.42
	Cumulative N balance (kg ha^−1^)	− 259 d	− 193 c	− 105 b	− 26 a
Kiboko	NRE (kg kg^−1^)		0.86	0.85	0.99
	Cumulative N balance (kg ha^−1^)	− 139 d	− 72 c	24 b	164 a
Harare	NRE (kg kg^−1^)		1.8 a	1.1 b	0.69 c
	Cumulative N balance (kg ha^−1^)	− 190 c	− 167 b	− 161 b	− 66 a

The NRE values are sourced from the Embu 2015SR, the average of Kiboko 2013LR and 2014SR, and Harare 2014/15 seasons. The cumulative N balance was summed over 9 seasons in Embu, 7 seasons in Kiboko, and 5 in Harare. Letters denote differences among the total plant or total soil contents at different N rates averaged across 6 hybrids as assessed by LSD ($\propto = 0.05$). Where there are no letters, the difference was not significant

The cumulative fertilizer N balance was negative at 0 kg N ha^−1^ and at the lowest non-zero N rate in all sites, as well as at the highest 2 N rates in Embu and Harare (Table 1). Had a third of the residue not been returned in Kiboko, the cumulative balance would have only reached a net positive balance of 14 kg N ha^−1^ in the highest N rate treatment plots (assuming the cumulative N uptake remained the same).

### Residual soil inorganic N

**Fig. 2:**
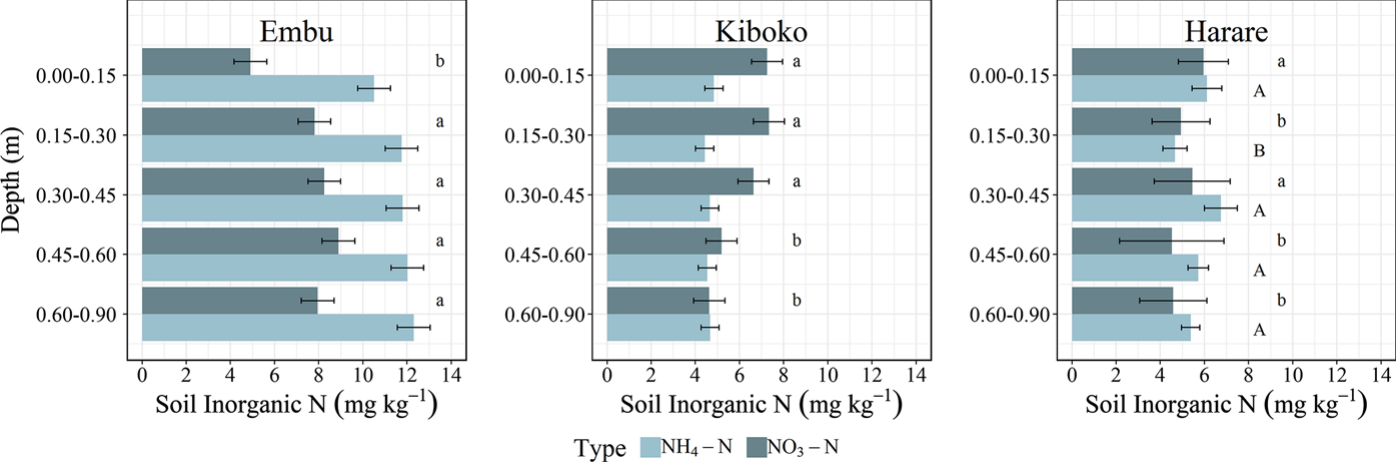
Soil inorganic N concentration (NH_4_-N and NO_3_-N at each depth increment in Embu, Kenya (post-season 2015SR), Kiboko, Kenya (post-season 2015SR), and Harare, Zimbabwe (post-season 2014/15). Letters denote differences among the NH_4_-N or NO_3_-N concentrations as assessed by LSD ($\propto = 0.05$). Where there are no letters, the difference was not significant. Error bars show the standard error of the mean

In Embu, there was no N rate effect on soil NH_4_-N or NO_3_-N concentrations (averaged across all depths) following harvest (Table 2). However, when total inorganic N content in the top 0.9 m of the plots was considered, slight but significant differences were detected among treatments: there was less inorganic N remaining in the soil where 30 kg N ha^−1^ was applied than where 0 or 90 kg N ha^−1^ were applied (Fig. 1). Nitrate-N concentration increased with depth, but there was no depth effect on NH_4_-N concentration (Fig. 2). There was no significant N rate by depth interaction effect on NO_3_-N and NH_4_-N concentrations or contents.

**Table 2:** Soil N pool concentrations averaged across all hybrids and depths in each site in Embu and Kiboko post-harvest 2015SR season and in Harare post-harvest 2014/15 season

Fertilizer N rate (kg ha^−1^)	NH_4_-N (mg kg^−1^)	NO_3_-N (mg kg^−1^)	Organic N (mg kg^−1^)	C:N
Embu				
0	12	6	2013 b	11.3
30	10	5	2137a	11.2
60	10	6	2096 a	11.3
90	11	6	2085 ab	11.2
Kiboko				
0	4	5 c	760	11.6 a
40	4	5 bc	856	10.3 b
80	4	6 b	827	12.4 a
160	3	8 a	780	11.8 a
Harare				
0	5	5	937	11.2
40	7	5	925	11.6
80	8	6	960	11.6
160	6	6	879	12.5

Letters by values denote significant differences among the treatments in a site as assessed by LSD ($\propto = 0.05$). Where there are no letters, the difference was not significant

In Kiboko, the average soil NO_3_-N concentration increased as N rate increased, but NH_4_-N concentration was unaffected by N rate (Table 2). Total inorganic N content in the top 0.9 m of soil also did not respond to N rate (Fig. 1). Soil NO_3_-N concentration decreased as the depth increased, but soil NH_4_-N concentrations were similar at all depth intervals (Fig. 2). There was no significant N rate by depth interaction effect on NO_3_-N and NH_4_-N concentrations or contents.

In Harare, a significant N rate by depth interaction effect on the soil NO_3_-N concentration was evident wherein the concentration increased with depth increments at 0 kg N ha^−1^, but accumulated more between 0.3 and 0.45 m relative to the other depth increments at the higher N rates (data not shown). The NH_4_-N concentration was lower in the 0.15–0.3, 0.45–0.6, and 0.6–0.9 m depth increments relative to the 0–0.15 and 0.3–0.45 m increments (Fig. 2). There was neither an N rate nor a N rate by depth interaction effect on average NH_4_-N concentrations. The total inorganic N content in the upper 0.9 m was greater in plots where 80 kg N ha^−1^ was applied relative to plots where 0 kg N ha^−1^ was applied, but otherwise N rates resulted in similar inorganic N contents (Fig. 1).

### Soil organic-nitrogen

While the distribution of inorganic N in the soil profile reflects the seasonal effects of applying N, differences in the organic-N reservoir reflect the long-term effects on crop N use and N loss from the soil.

In Embu, the organic-N concentration was higher at 30 and 60 kg N ha^−1^ relative to that at 0 kg N ha^−1^ (Table 2). In Kiboko and Harare, the average concentrations of organic N across the top 0.9 m (Table 2) were unaffected by N rate treatments. In all sites, averaged across all N rates, organic-N concentrations decreased as depth increased (data not shown).

The total soil C to total soil N ratio (C:N) in the 3 sites in the top 0.15 m (where C:N is typically measured and is most likely to reflect the soil’s OM and microbial biomass levels) varied in their responses to N rate. In the top 0.15 m, the soil C:N in Embu, averaged across all N rates, was 11.3:1; it was lower at 30 and 90 kg N ha^−1^ than at 0 kg N ha^−1^ (Table 2). In Kiboko, averaged across all N rates, the C:N in the top 0.15 m was 12.5:1; it was lower at 40 kg N ha^−1^ than at 0, 80, and 160 kg N ha^−1^, but did not differ among the latter 3 N rates (Table 2). In Harare, the C:N in the top 0.15 m, averaged across all N rates, was 11.7:1; it was unaffected by N rate (Table 2).

**Fig. 3:**
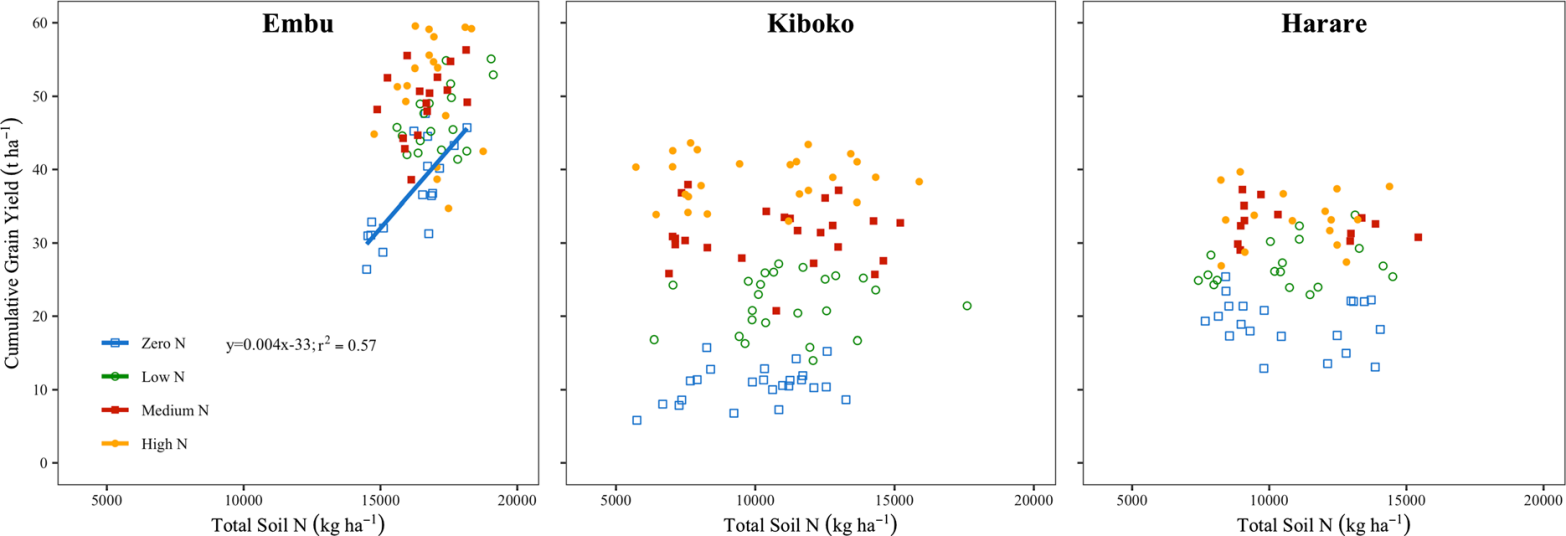
Total soil N content to a depth of 0.9 m and cumulative grain yield averaged across all hybrids in Embu, Kenya (2012LR–2015SR, totaling 9 consecutive seasons), Kiboko, Kenya (2011LR–2014LR seasons, totaling 7 consecutive seasons with one season of data missing from 2012SR), and Harare, Zimbabwe (2010/11–2014/15, totaling 5 consecutive seasons) at each N rate (Zero N, Low N, Medium N, and High N referring to 0, 30, 60, and 90 kg N ha^−1^ in Embu and 0, 40, 80, and 160 kg N ha^−1^ in Kiboko and Harare, respectively). The relationship between soil N content and cumulative yield was significant (*P *≤ 0.05) in Embu at 0 kg N ha^−1^, but not in the other sites or at the other N rates. A regression line was therefore fitted to the data points from that site/treatment, but not the others

While there were relatively strong correlations between plant N content and grain yield (Supplementary Figure 1; Embu: r = 0.82; Kiboko: r = 0.91; Harare: r = 0.99), Embu was the only site where there was a significant relationship between cumulative grain yield and total soil N (Fig. 3). In Embu, at 0 kg N ha^−1^, cumulative grain yield increased at a rate of 4 kg ha^−1^ for every additional kg ha^−1^ of soil N content in the top 0.9 m (Fig. 3). Cumulative yield in Embu increased at a rate of 18 kg ha^−1^ for every additional kg ha^−1^ of soil N content in the top 0.15 m at both 0 and 90 kg N ha^−1^ (r^2^ = 0.51 and 0.21, respectively; data not shown). There was no relationship between cumulative yield and soil N contents (in the top 0.9 or 0.15 m) at the other N rates.

## Discussion

### Plant-induced soil N depletion

The NRE values found in this study fell within the range or above those found in other field experiments in SSA. Three other studies in SSA calculated NRE at 120 kg N ha^−1^: Nyamangara et al. (2003) looked at the NRE values of local hybrids in Zimbabwe, Kurwakumire et al. (2014) investigated NRE for hybrid SC513 in Zimbabwe, and Oikeh et al. (2003) determined the NRE values of five tropical maize hybrids, four of which were selected for enhanced root growth capacity while one was an open pollinated control, in Nigeria. These 3 studies found NRE values ranging from 0.31 to 0.69 kg kg^−1^, a range consistent with the values found in Embu at all N rates and in Harare at 160 kg N ha^−1^. Higher NRE values, like those found in Kiboko at all N rates and Harare at the lower two non-zero N rates (40 and 80 kg N ha^−1^) have also been found to some extent in SSA. In Nigeria, Akintoye et al. (1999) found NRE values of a wide variety of single- and double-cross maize hybrids averaged over N rates of 70, 140, and 210 kg N ha^−1^ to range from 1.06 to 1.35 kg kg^−1^. Similarly, at 60 kg N ha^−1^ (Nyamangara et al. 2003) and 90 kg N ha^−1^ (Oikeh et al. 2003), NRE values were as high as 1.0 kg kg^−1^ and 1.63 kg kg^−1^, respectively. The range of NRE values found in Kiboko at all N rates and in Harare at 80 kg N ha^−1^ fell within the higher range of NRE values found in Akintoye et al. (1999), Nyamangara et al. (2003), and Oikeh et al. (2003). When 40 kg N ha^−1^ was applied in Harare, however, the NRE value spiked to 1.8 kg kg^−1^, exceeding all values found in the literature. We believe that this exceptionally high NRE value is not an anomaly, but rather, points to the potential of newer maize hybrids to further deplete the soil of inorganic N at lower N rates (Mueller et al. 2019).

A review by Ciampitti and Vyn (2012) found that, generally, NRE decreases as N rate increases. In our study, the only site in which NRE increased when N rate decreased was Harare. Embu and Kiboko’s stagnant NRE values suggest that while N uptake was likely restricted by drought stress in Embu and sub-optimal N rates in Kiboko (Pasley et al. 2019), these constraints lessened with increased fertilizer N supply (Vanlauwe et al. 2001; Betrán et al. 2003). Meanwhile, P deficiency in both Embu and Harare likely equally limited N uptake at all N rates (Pasley et al. 2019). Like Harare, some other studies in SSA showed NRE values increased as the N rate decreased, but unlike Harare, they did not apply such a low fertilizer N rate as 40 kg N ha^−1^. As acknowledged previously, a low rate of 40 kg N ha^−1^ is more representative of what is typically applied in SSA than the higher N rates. Our findings in Harare, therefore, suggest that maize in a typical small-holder farm in SSA may be taking up almost two-fold the amount of N applied, with the more than half arising from N depletion of soil reserves.

### Mitigating soil N depletion with inorganic N fertilizer

In general, the cumulative N balance range found in Embu and Harare was negative at all N rates; this is consistent with previous studies examining maize hybrid response to N fertilizer in West Africa (Oikeh et al. 2003). Consistent with their higher NRE values, hybrids in Kiboko had an average annual fertilizer N balance of − 37 kg N ha^−1^ across all N rates applied. Had no stover residue been returned to the soil post-harvest for the same hybrids in Kiboko, an average fertilizer N balance of − 33, − 32, and 13 kg N ha^−1^ for N rates of 40, 80, and 160 kg N ha^−1^, respectively, would have resulted. These average annual depletion rates were significantly higher than those of Embu (− 29, − 21, − 12, and − 3 kg N ha^−1^ at 0, 30, 60, and 90 kg N ha^−1^, respectively) (*P *< 0.05). Oikeh et al. (2003) found less residual inorganic N in the top 0.9 m when they had higher NRE values in maize production. Similarly, the higher NRE values in Kiboko and Harare, relative to Embu, may, in part, explain why Embu had more inorganic N in the top 0.9 m than the other 2 sites (*P *< 0.05). Hybrid selection in our study did not impact the fertilizer N balance, a conclusion also reached by Oikeh et al. (2003).

In clay-rich Embu and Harare, the soil NO_3_-N and/or NH_4_-N increased in at least one of the subsoil depths relative to shallower depths (data not shown). In Embu, this increase in inorganic N was due to increases in both NO_3_-N and NH_4_-N, but in Harare this increase was solely a result of increased NH_4_-N at the 0.3–0.45 m depth increment. This retention of inorganic N, especially at the deeper depths, was also found in other studies on acidic soils where the anion exchange capacity of kaolinite reduced NO_3_-N leaching (Thomas and Hargrove 1984; Oikeh et al. 2003; Xie et al. 2018). In general, consistent with the aforementioned negative N balance values, soil inorganic N levels in all sites were low and, therefore, the potential for N leaching was also low. Higher levels of exchangeable acidity may have restricted nitrification (Kemmitt et al. 2006; Nugroho et al. 2007; Zhao et al. 2017), resulting in the accumulation of NH_4_-N throughout the soil profile in Embu and at the 0.3–0.45 m depth increment in Harare.

While both Embu and Harare have clay-rich soils, which tend to protect soil organic N from decomposition by heterotrophic microorganisms (Christensen 1996; Mungai et al. 2005), the dry conditions in Embu may also have depressed microbial activity and, thus, soil organic N turnover in contrast to the better-watered conditions of Kiboko and Harare (Stanford and Epstein 1974; Hart et al. 1994; Vanlauwe et al. 2015). Nevertheless, there was a positive correlation between cumulative grain yield over the 9 growing seasons and total soil N to a depth of 0.9 m at 0 kg N ha^−1^ in Embu, suggesting that grain yield was still impacted more by mineralization of organic N at 0 kg N ha^−1^ in this site than in the other two sites. Soil OM levels were higher in Embu than in Kiboko and Harare; this suggests higher levels of labile organic N available for mineralization in Embu. While soil N mineralization probably also occurred in Kiboko and Harare, these two sites also had a greater tendency towards N being recalcitrant as there was less total organic N in the rooting profile. Embu soil’s low moisture and pH characteristics may have also limited how much additional N was lost to leaching (Tully et al. 2016).

## Conclusion

While the application of inorganic N fertilizer alone did not mitigate or lessen soil N depletion, the findings of this study point to the potential of the integrated application of inorganic and organic N fertilizer to increase maize yields in low N sites without exacerbating soil N depletion. Positive soil N responses to N fertilizers may not be seen, however, at N rates as low as 40 kg N ha^−1^. More research is needed to define the optimal application rates of inorganic and organic N needed for grain and whole-plant maize production across variable environments. Such future work should include sampling soil below a 0.3 m depth, as this study found that soil N was accumulated deeper in the profile. In general, our findings suggest that advances in breeding for higher yields and more N use efficient hybrids may have negative consequences for soil N status unless more research effort is invested in finding practical and effective N management strategies for SSA maize production at low to moderate N input levels.

## Electronic supplementary material

Below is the link to the electronic supplementary material.

Supplementary material 1 (TIFF 54931 kb)

Supplementary material 2 (DOCX 34 kb) 
