# Supplementary figures and images for: Nitrogen rate impacts on tropical maize nitrogen use efficiency and soil nitrogen depletion in eastern and southern Africa
Source: Nutr Cycl Agroecosyst. 2020 Feb 13;116(3):397–408. doi: 10.1007/s10705-020-10049-x (PMC7380447; doi:10.1007/s10705-020-10049-x)

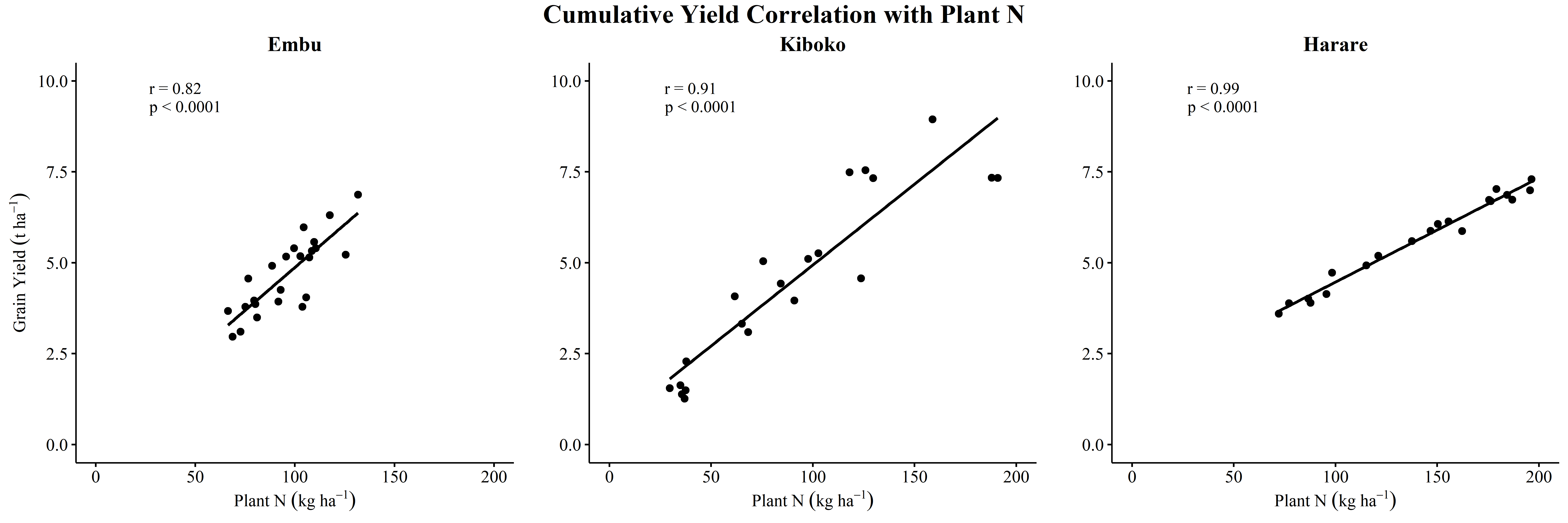

Supplement: Supplementary file 1 — Supplementary material 1 (TIFF 54931 kb) [file 10705_2020_10049_MOESM1_ESM.tiff]
