# Supplementary material for: Nitrogen rate impacts on tropical maize nitrogen use efficiency and soil nitrogen depletion in eastern and southern Africa
Source: Nutr Cycl Agroecosyst. 2020 Feb 13;116(3):397–408. doi: 10.1007/s10705-020-10049-x (PMC7380447; doi:10.1007/s10705-020-10049-x)
Supplement: Supplementary file 2 — Supplementary material 2 (DOCX 34 kb) [file 10705_2020_10049_MOESM2_ESM.docx]

*Supplementary Table 1a. Soil characteristics of Embu (FAO soil classification, texture, bulk density (BD), %Organic Matter (OM), C:N, amorphous Fe, cation exchange capacity (CEC), pH (1:1 soil water slurry), and exchangeable acidity.*

| **Depth (m)** | **FAO Soil Classification** | **Texture** | **BD (Mg m^-3^)** | **OM (%)** | **C:N** | **Amorph. Fe** | **CEC** | **pH** | **Exch Acidity** |
| --- | --- | --- | --- | --- | --- | --- | --- | --- | --- |
|  |  |  |  |  |  | **(mg kg^-1^)** | **(meq 100g^-1^)** |  | **(meq 100g^-1^)** |
| 0-0.15 | Humic Nitisol | Clay Loam | 0.93 | 5.79 | 11 | 16.32 | 11.89 | 5.15 | 3.51 |
| 0.15-0.3 |  | Clay | 1.03 | 5.52 | 11 | 16.26 | 12.35 | 5.12 | 3.51 |
| 0.3-0.45 |  | Clay | 0.95 | 5.15 | 12 | 15.99 | 12.59 | 5.24 | 3.51 |
| 0.45-0.6 |  | Clay Loam | 0.94 | 4.00 | 11 | 15.99 | 10 | 5.42 | 3.51 |
| 0.6-0.9 |  | Clay Loam | 0.92 | 3.21 | 11 | 19.32 | 8.18 | 5.46 | 1.75 |

*Supplementary Table 1b. Soil characteristics of Kiboko (FAO soil classification, texture, bulk density (BD), %Organic Matter (OM), C:N, amorphous Fe, cation exchange capacity (CEC), and pH (1:1 soil water slurry)).*

| **Depth (m)** | **FAO Soil Classification** | **Texture** | **BD (Mg m^-3^)** | **OM (%)** | **C:N** | **Amorph. Fe** | **CEC** | **pH** |
| --- | --- | --- | --- | --- | --- | --- | --- | --- |
|  |  |  |  |  |  | **(mg kg^-1^)** | **(meq 100g^-1^)** |  |
| 0-0.15 | Acri-Rhodic Ferrasols | Sandy Loam | 1.53 | 2.61 | 12 | 5.35 | 9.44 | 7.82 |
| 0.15-0.3 |  | Sandy Clay Loam | 1.49 | 2.28 | 12 | 5.36 | 7.57 | 7.63 |
| 0.3-0.45 |  | Sandy loam | 1.42 | 2.08 | 12 | 4.93 | 8.11 | 7.64 |
| 0.45-0.6 |  | Sandy loam | 1.47 | 1.70 | 11 | 5.4 | 6.47 | 7.28 |
| 0.6-0.9 |  | Sandy Clay Loam | 1.49 | 1.53 | 12 | 5.29 | 6.66 | 7.07 |

*Supplementary Table 1c. Soil characteristics of Harare (FAO soil classification, texture, bulk density (BD), %Organic Matter (OM), C:N, amorphous Fe, cation exchange capacity (CEC), pH (1:1 soil water slurry), and exchangeable acidity.*

| **Depth (m)** | **FAO Soil Classification** | **Texture** | **BD (Mg m^-3^)** | **OM (%)** | **C:N** | **Amorph. Fe** | **CEC** | **pH** | **Exch Acidity** |
| --- | --- | --- | --- | --- | --- | --- | --- | --- | --- |
|  |  |  |  |  |  | **(mg kg^-1^)** | **(meq 100g^-1^)** |  | **(meq 100g^-1^)** |
| 0-0.15 | Ferric Luvisols | Clay Loam | 1.37 | 2.59 | 12 | 13.38 | 7.82 | 5.59 | 2.85 |
| 0.15-0.3 |  | Clay | 1.38 | 1.95 | 12 | 14.49 | 8.48 | 5.73 | 1.75 |
| 0.3-0.45 |  | Clay | 1.32 | 2.05 | 12 | 12.78 | 9.52 | 5.82 | 2.63 |
| 0.45-0.6 |  | Clay | 1.3 | 1.36 | 12 | 12.09 | 7.51 | 5.88 | 1.32 |
| 0.6-0.9 |  | Clay | 1.34 | 1.42 | 12 | 9.75 | 8.21 | 5.9 | 1.46 |

*Supplementary Table 2. Site management history and details and timeline of data collection.*

| Management | | | | | | | | Timing of Data Collection | | |
| --- | --- | --- | --- | --- | --- | --- | --- | --- | --- | --- |
| 5 Season History | Date Established | N Rate  kg ha^-1^ | N Source | P Rate  kg ha^-1^ | P Source | Population plants ha^-1^ | Row Spacing | Grain Yield | Whole Plant Biomass | For Soil Analysis |
| **Embu** | | | | | | | | | | |
| Cont. Maize with no inputs | 2011SR | Seasons 1-4: 0, 40, 80, 120; 5-9: 0, 30, 60, 90 | Calcium Ammonium Nitrate (33.5% N) | 20 | Triple Super Phosphate (20%) | 53,300 | 0.75 m | Every Season | Harvest 2015SR | Post-Harvest 2015SR |
| **Kiboko** | | | | | | | | | | |
| Cont. Sorghum (Sorghum bicolor (L.) ) with no inputs | 2011LR | Seasons 1-2: 0, 40, 80, 120; 3-7: 0, 40, 80, 160 | Calcium Ammonium Nitrate (33.5% N) | 20 | Triple Super Phosphate (20%) | 44,400 | 0.75 m | Every Season | Harvest 2013SR/2014LR | Post-Harvest 2015SR |
| **Harare** | | | | | | | | | | |
| Cont. Maize with no inputs | 2010/2011 | All Seasons: 0, 40, 80, 160 | Ammonium Nitrate (26%) | 20 | Triple Super Phosphate (20%) | 66,700 | 0.75 m | Every Season | Harvest 2014/15 | Post-Harvest 2014/15 |

*Supplemental Table 3. Hybrid name, ID, manufacturer, maturity classification (cutoff between early and Late maturity is 68 days from planting date to anthesis date (AD)), and year of coding (for CIMMYT hybrids)/release (for commercial hybrids) for each site in Embu 2013SR to 2015SR seasons, Harare 2013/14 and 2014/15 seasons, and Kiboko 2012LR to 2014SR seasons.*

| **ID** | **Hybrid Name** | **Manufacturer** | **Maturity Classification** | **Year of Coding/Release** |
| --- | --- | --- | --- | --- |
|  |  |  | **(68 days to AD cutoff between Early and Late)** |  |
| **Embu** | | | | |
| 1 | PHB3253 | DuPont Pioneer | Late | 1996 |
| 2 | DK8031 | Monsanto | Late | 2003 |
| 3 | PAN4M-19 | Pannar Seed | Late | 2008 |
| 4 | Duma43 | Seed Co Ltd | Late | 2004 |
| 5 | H513 | Kenya Seed Co | Late | 1995 |
| 6 | WH403 | Western Seed Co | Late | 2003 |
| **Harare** | | | | |
| 1 | CZH132085 | CIMMYT | Late | 2013 |
| 2 | TH127618 | CIMMYT | Late | 2012 |
| 3 | CZL1242 | CIMMYT | Late | 2012 |
| 4 | SC403 | Seed Co Ltd | Early | 1998 |
| 5 | SC513 | Seed Co Ltd | Early | 1997 |
| 6 | PAN413 | Pannar Seed | Late | 1998 |
| **Kiboko** | | | | |
| 1 | WH507 | Western Seed Co | Early | 2006 |
| 2 | CZH0616 | CIMMYT | Early | 2006 |
| 3 | CIM1 | CIMMYT | Early | 2008 |
| 4 | CIM2 | CIMMYT | Early | 2008 |
| 5 | CKH101572 | CIMMYT | Late | 2010 |
| 6 | H513 | Kenya Seed Co | Early | 1995 |

*Supplementary Table 4. Rainfall, average temperature for season, maximum daily temperature during critical period (CP), minimum daily temperature during the grain filling period (GF), and daytime and estimated nighttime temperature during the GF for 2013SR-2015SR seasons in Embu, 2012SR-2013SR seasons in Kiboko, 2013/14 and 2014/15 seasons in Harare. “LR” stands for long rains season and “SR” stands for short rains season. Season length is determined as days to black layer which was estimated as twice the number of days from planting to average 50% silking unless crop was harvested prior to this day in which case, the harvest date was used to calculated season length. The CP was defined as the 4 week window around the anthesis-silking interval (ASI), the GF was defined as the time interval between the CP and black layer/harvest. Minimum nighttime temperature during the GF was estimated by the first quartile of the daily temperatures during the GF and the maximum daytime temperature during the GF was estimated by the third quartile of the daily temperatures during the GF.*

|  |  |  | **Rainfall (mm)** | | |  | | | **Temperature ˚C** | | | |
| --- | --- | --- | --- | --- | --- | --- | --- | --- | --- | --- | --- | --- |
| **Site** | **Season** | **Season Length (Days)** | **Season** | **CP** | **GF** | **Average Season** | **CP Max** | **GF Min** | | **GF Q1** | **GF Q3** |  |
| **Embu** | **2013 SR** | 157 | 257 | 57 | 7 | 19.7 | 20.5 | 19.3 | | 20.3 | 21.8 |  |
|  | **2013 LR** | 167 | 128 | 9 | 18 | 19.3 | 20.1 | 16.9 | | 18.3 | 19.9 |  |
|  | **2014 SR** | 153 | 187 | 0 | 74 | 19.8 | 20.7 | 19.0 | | 20.1 | 21.3 |  |
|  | **2014 LR** | 153 | 104 | 20 | 67 | 20.3 | 21.2 | 18.2 | | 19.4 | 20.3 |  |
|  | **2015 SR** | 145 | 132 | 3 | 31 | 20.2 | 20.6 | 19.3 | | 21.0 | 21.9 |  |
| **Harare** | **2013/14** | 161 | 556 | 64 | 63 | 18.8 | 21.1 | 13.4 | | 15.4 | 18.1 |  |
|  | **2014/15** | 161 | 567 | 56 | 70 | 19.2 | 21.7 | 14.3 | | 16.8 | 18.6 |  |
| **Kiboko** | **2013 SR** | 159 | 50 | 2 | 41 | 27.9 | 29.6 | 24.9 | | 28.1 | 29.4 |  |
|  | **2013 LR** | 128 | 360 | 7 | 278 | 27.9 | 30.2 | 24.0 | | 25.9 | 28.1 |  |
|  | **2014 SR** | 159 | 53 | 23 | 18 | 27.0 | 28.1 | 26.0 | | 27.5 | 28.8 |  |
|  | **2014 LR** | 128 | 129 | 15 | 85 | 28.4 | 30.4 | 24.6 | | 27.2 | 29.5 |  |
